# Thermal conductivity and conductance of protein in aqueous solution: Effects of geometrical shape

**DOI:** 10.1002/jcc.27048

**Published:** 2022-12-05

**Authors:** Ikuo Kurisaki, Seiya Tanaka, Ichiro Mori, Toshihito Umegaki, Yoshiharu Mori, Shigenori Tanaka

**Affiliations:** ^1^ Graduate School of System Informatics Kobe University Kobe Japan; ^2^ Graduate School of Science, Technology and Innovation Kobe University Kobe Japan; ^3^ Center for Mathematical Modeling and Data Science Osaka University Osaka Japan

**Keywords:** hydrated protein, molecular dynamics, salt addition, thermal conductance, thermal conductivity

## Abstract

Considering the importance of elucidating the heat transfer in living cells, we evaluated the thermal conductivity *κ* and conductance *G* of hydrated protein through all‐atom non‐equilibrium molecular dynamics simulation. Extending the computational scheme developed in earlier studies for spherical protein to cylindrical one under the periodic boundary condition, we enabled the theoretical analysis of anisotropic thermal conduction and also discussed the effects of protein size correction on the calculated results. While the present results for myoglobin and green fluorescent protein (GFP) by the spherical model were in fair agreement with previous computational and experimental results, we found that the evaluations for *κ* and *G* by the cylindrical model, in particular, those for the longitudinal direction of GFP, were enhanced substantially, but still keeping a consistency with experimental data. We also studied the influence by salt addition of physiological concentration, finding insignificant alteration of thermal conduction of protein in the present case.

## Graphical abstract

Thermal conductivity and conductance for hydrated protein were evaluated through molecular dynamics simulations under the periodic boundary condition. In addition to the spherical approximation to the protein shape, we considered the cylindrical model in which the size of protein was appropriately estimated. The dependences of the calculated results on the protein shape and the simulation box size were carefully analyzed.

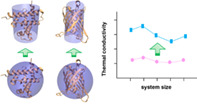

## 1 INTRODUCTION

Temperature is a basic physico‐chemical parameter to regulate biological phenomena. Examples include catalytic activities of enzymes,^1^, ^2^, ^3^, ^4^ thermosensitive channels,^5^ structural stabilities of proteins and nucleic acids,^6^ and rate constants of charge or energy transfer^7^, ^8^, ^9^ at molecular level. However, in spite of its significance, the elucidation of the behaviors and roles of temperature and heat remains elusive in the contexts of molecular and cellular biology.

Recently, there appeared some studies for the local measurements of temperature in living cells, where the existence of the microscopic inhomogeneity of temperature distribution was reported.^10^, ^11^, ^12^, ^13^, ^14^, ^15^, ^16^ This remarkable observation has suggested a possibility that the intracellular temperature may play an important role as a biological signal, while a number of controversial issues such as “10^5^ gap problem”^12^, ^13^, ^14^, ^15^ concerning the temperature relaxation remain. We then need further microscopic understanding of the biological roles of temperature and heat transfer at molecular level, whereas the experimental observations on the relationship between temperature and biomolecular behaviors are still difficult in spite of recent advances in experimental technologies.^17^, ^18^, ^19^

As a whole, we have very limited information about the thermal conduction and temperature relaxation associated with solvated biomolecules.^20^, ^21^, ^22^, ^23^ Hence the use of molecular simulations may be promising to elucidate the microscopic mechanisms of thermal conduction at intracellular level. Substantial investigations have been carried out experimentally and computationally for both lipid membranes^24^, ^25^, ^26^, ^27^, ^28^, ^29^ and proteins.^17^, ^18^, ^19^, ^30^, ^31^, ^32^, ^33^, ^34^, ^35^, ^36^, ^37^ Lipid membranes are usually in solid‐like state in the cell, thus being relatively easily investigated by conventional experimental and computational methods.^20^ Meanwhile, relatively small portion of proteins takes solid‐like form under specific conditions but most of proteins are usually dispersed in the aqueous solution. Thus, the conventional experimental methods, which employ solid‐like materials for observations, are not applicable to estimate the thermal conductivity and conductance under the hydrated conditions, where proteins often express their biochemical functions. We then focus on the computational studies regarding the thermal conductivity and conductance of hydrated proteins in this work.

In the present study, we pay a special attention to a pioneering simulation work performed by Lervik et al.^31^ in which the authors calculated the thermal conductivity and conductance of various proteins by fitting the data on the temperature relaxation of hydrated protein obtained through non‐equilibrium molecular dynamics (MD) simulation to the heat diffusion equation. Although their calculated results agreed well with known experimental data, they assumed that the protein had a spherical shape and employed the water sphere to solvate the protein. We may, however, remark that actual proteins or their complexes often have non‐spherical shapes including extreme cases of disk or wire like shapes. We may then expect the anisotropic thermal conduction in these cases and the use of solvated water sphere would bring about some inefficiencies and inaccuracies in simulations. Hence, in the present study, we extend their computational scheme so as to consider the cylindrical model for protein in addition to the spherical model along with the use of the periodic boundary condition for the hydrated system. Besides, in projecting the MD data for the temperature relaxation of protein onto the heat diffusion equation, the estimation of protein size is crucial for the accurate evaluation of thermal conductivity (inside protein) and thermal conductance (at protein‐water interface). While Lervik et al.^31^ estimated the radius of a spherical protein in terms of its gyration radius, we see their protocol would bring about some overestimations (in the case of spherical protein) or underestimations (in the case of cylindrical protein) for the evaluations of thermal conductivity and conductance. We investigate this issue in details and propose some procedures to correct these errors. Furthermore, we study the effects of salt addition on the thermal conduction of protein by performing the MD simulations for protein in aqueous solution containing NaCl ions, which would provide some insights into the molecular crowding effects in cells.

In the following Materials and Methods section, we illustrate the computational procedures employed in the present study. Some additional details are complemented with Supporting Information. Section 3 is devoted to addressing the Results and Discussion in which the results obtained with the spherical and cylindrical models are comprehensively compared for myoglobin (Mb) and green fluorescent protein (GFP), along with the issue of salt addition. Section 4 concludes with summary.

## 2 MATERIALS AND METHODS

### 2.1 Molecular dynamics simulations

**FIGURE 1:**
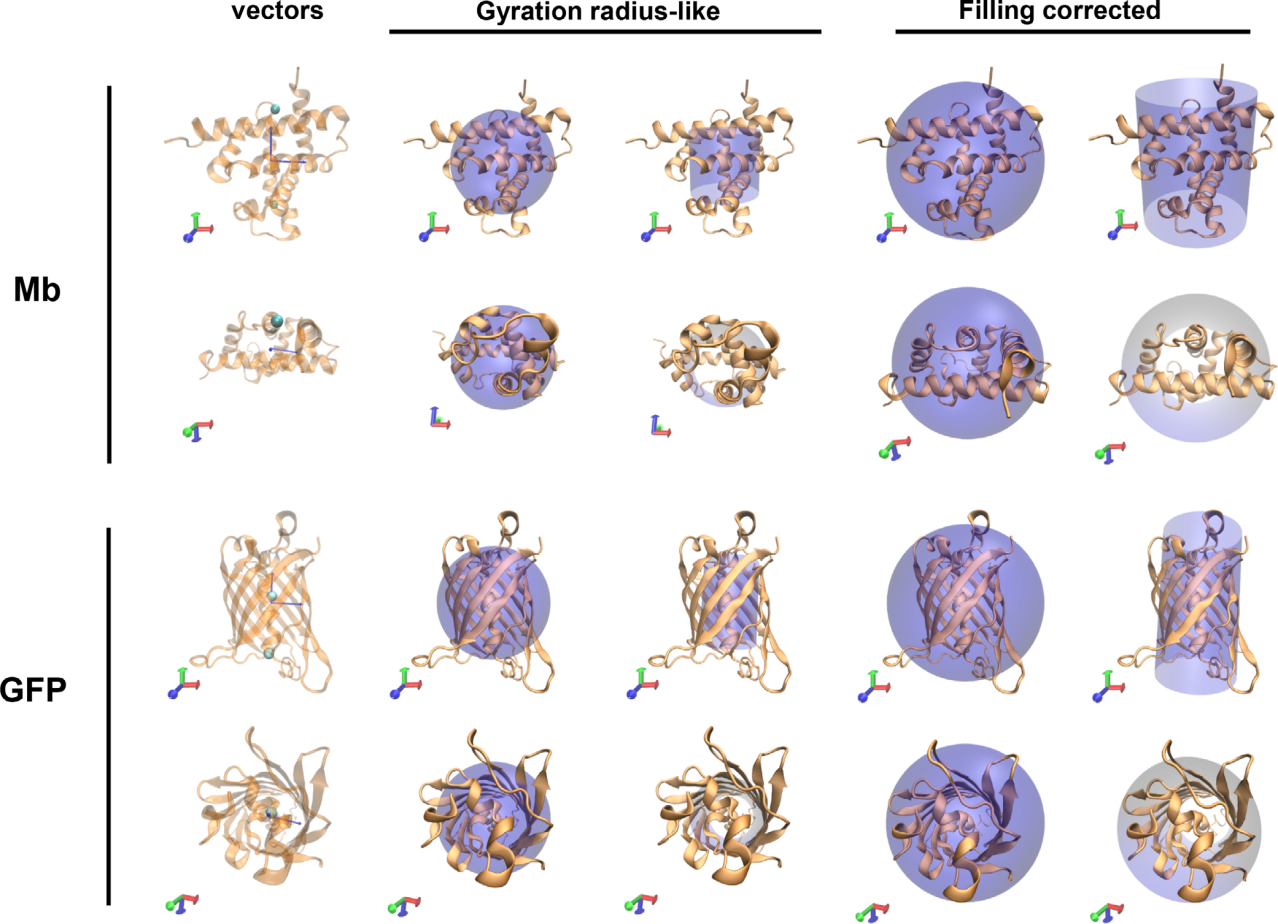
Molecular structures of myoglobin (Mb) and green fluorescent protein (GFP) by side (upper panel) and top (lower panel) views. Two vectors represented by arrows along the radial and longitudinal directions in cylindrical coordinates are assigned (left panel). Geometrical relations between actual protein structures and approximate sphere or cylinder models (colored in purple) are also illustrated when the protein sizes are described in terms of gyration radius like (middle panel) or fully filling (right panel) lengths.

By means of all‐atom molecular dynamics (MD) simulations, we have examined two proteins: one is myoglobin^38^ (Mb, PDB entry: 1MBS) and the other is green fluorescent protein (GFP, PDB entry: 1QXT). As illustrated in Figure 1, the former and latter have approximately globular (spherical) and cylindrical shapes, respectively.

The atomistic interactions were modeled by using the GROMOS96 43a2 force field.^39^ The X‐ray crystal structure of the protein was solvated in the rectangular box with the simple point charge‐extended model (SPC/E)^40^ for water molecules and electrically neutralized by adding counter ions. For each of Mb and GFP, we examined five cases of system size and two cases (pure water or salt addition) of ion concentration. The system components are summarized in Table 1. It is noted that the system size is described by the distance from the box edge to solute (protein) molecules along *x*, *y* and *z* axis, which is set for water to sufficiently solvate the solute in the rectangular box.

**TABLE 1:** Settings of MD simulations for hydrated proteins, myoglobin (Mb) and green fluorescent protein (GFP), where simulation box size (see the text) and numbers of water molecules, Na^+^ and Cl^−^ ions contained in the simulation cell are shown in the cases of (A) pure water and (B) 140 mM NaCl aqueous solution systems

Box size (nm)	# of water	# of Na^+^	# of Cl^−^	# of water	# of Na^+^	# of Cl^−^
(A)	Mb in water			GFP in water		
1.0	10,126	0	3	13,331	8	0
2.0	22,600	0	3	28,581	8	0
3.0	41,941	0	3	51,290	8	0
4.0	69,507	0	3	82,394	8	0
4.5	87,774	0	3	101,845	8	0
(B)	Mb in NaCl solution			GFP in NaCl solution		
1.0	10,070	28	31	13,235	46	38
2.0	22,480	60	63	28,429	84	76
3.0	41,721	110	113	51,022	142	134
4.0	69,143	182	185	81,964	223	215
4.5	87,318	228	231	101,313	274	266

Each protein system was energetically minimized by using the steepest descent method. Then, the system temperature of the whole system was equilibrated under NVT condition (300 K) for 100 ps, and the system density was relaxed by 1 ns‐NPT simulation (300 K, 1 bar). After these initial equilibrations, the protein and the remaining of the system were coupled with independent Berendsen thermostats,^41^ whose temperatures were set to 400 and 300 K, respectively. Under such a dual thermostat condition, the system temporarily ran for 10 ps, which was sufficient to generate temperature difference between the protein and the remaining. After this equilibration process, the thermostat on the protein was removed and then the system was relaxed for 100 ps. We repeated the above MD simulation 10 times for each of the systems and performed the following temperature relaxation analyses (see Section 2.2).

Through all the simulations, we employed the Berendsen thermostat^41^ with the coupling constant of 0.1 ps, following the earlier study by Lervik et al.^31^ Pressure of the system was regulated by using the Parrinello‐Rahman barostat^42^ with the coupling constant of 2 ps. The barostat was steadily applied to the system after the 1 ns‐NPT simulation. All chemical bonds including the hydrogen atom in the proteins and water were kept rigid using the LINCS algorithm. Nonbonded interactions were treated with the Particle Mesh Ewald algorithm. The real‐space cutoff values for both of the van der Waals interactions and the Coulombic interactions were set to 10 Å. Time step for the integration of equation of motion was set to 2 fs. All simulations and molecular modellings were performed using the GROMACS simulation package (version 5.1.5).^43^

### 2.2 Temperature relaxation analyses

**FIGURE 2:**
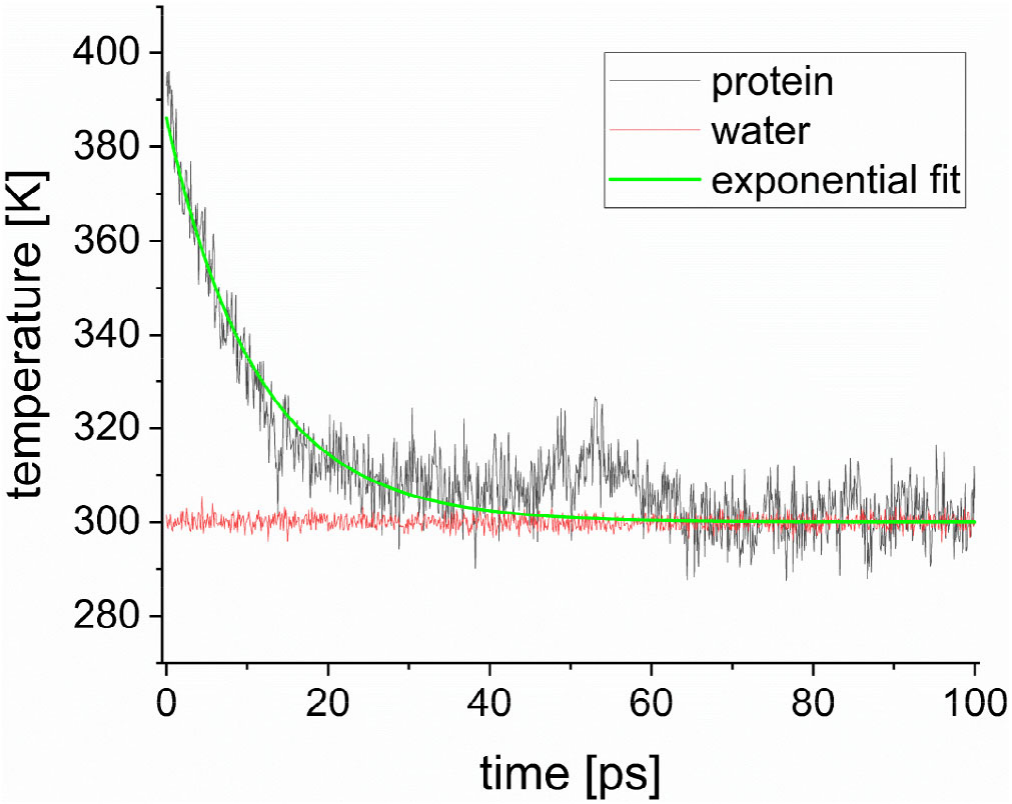
Example for the behavior of spatially averaged, time‐dependent temperature of protein, *T*(*t*), for the case of Mb in the water box of 1 nm size (black curve). Temporal change of water temperature (red curve) and the exponential fitting to the protein temperature (green curve) are also illustrated.

To estimate thermal conductivity (*κ* [W/K/m]) inside the protein and thermal conductance (*G* [MW/K/m^2^]) at the protein‐water interface, an MD‐derived temperature relaxation curve (see Figure 2 below) was fitted to a solution of the heat diffusion equation (HDE)^44^, ^45^:

(1) $$\frac{\partial T}{\partial t}=D\Delta T=D\left(\frac{\partial^{2}}{\partial x^{2}}+\frac{\partial^{2}}{\partial y^{2}}+\frac{\partial^{2}}{\partial z^{2}}\right)T$$

where *T* is the spatiotemporally varying temperature (associated with kinetic energy) of protein and *D* refers to the thermal diffusion constant.

As in the case of the previous study,^31^ the temperature relaxation was described as a simple exponential decay. Besides the HDE with spherical symmetry, which was discussed in the previous study,^31^ we newly considered the solution of HDE with cylindrical symmetry, thus accounting for the dependence of thermal conduction properties on protein shape.

Equation (2) below is the solution of HDE for the time‐dependent temperature of protein obtained via spatial averaging with the spherical coordinate for radius direction^31^:

(2) $$\frac{T\left(t\right)-T_{f}}{T_{i}-T_{f}}=\tilde{T}\left(t\right)\approx 6\frac{1}{{\lambda_{1}}^{3}}\frac{{\left(\sin\left(\lambda_{1}\right)-\lambda_{1}\cos\left(\lambda_{1}\right)\right)}^{2}}{\left(\lambda_{1}-\sin\left(\lambda_{1}\right)\cos\left(\lambda_{1}\right)\right)}\exp\left(\frac{-{\lambda_{1}}^{2}t}{\tau }\right)$$

where only the first term with respect to the *n* series (λ_n_ expansion)^31^ is retained (single exponential approximation).

Meanwhile, Equations (3a) and (3b) below are the spatially averaged solutions of HDE obtained with the cylindrical coordinates for radius (radial) and height (longitudinal or vertical) directions, respectively using the single exponential approximation:

(3a) $$\frac{T\left(t\right)-T_{f}}{T_{i}-T_{f}}=\tilde{T}\left(t\right)\approx 4\frac{1}{{\lambda_{1}}^{2}}\frac{J_{1}{\left(\lambda_{1}\right)}^{2}}{J_{0}{\left(\lambda_{1}\right)}^{2}+J_{1}{\left(\lambda_{1}\right)}^{2}}\exp\left(\frac{-{\lambda_{1}}^{2}t}{\tau }\right)$$

(3b) $$\frac{T\left(t\right)-T_{f}}{T_{i}-T_{f}}=\tilde{T}\left(t\right)\approx \frac{4\cdot \sin^{2}\left(\lambda_{1}\right)}{\lambda_{1}\cdot \left(2\lambda_{1}+\sin\left(2\lambda_{1}\right)\right)}\exp\left(\frac{-{\lambda_{1}}^{2}t}{\tau }\right)$$

Here, *T*_*i*_ and *T*_*f*_ are the initial temperature of the protein and the reference temperature for the remaining system, respectively. $T\left(t\right)$ is the spatially averaged temperature of the protein at the time *t*. In Equation (3a), $J_{0}\left(•\right)$ and $J_{1}\left(•\right)$ denote the 0th and 1st order (first kind) Bessel functions, respectively. The temperature, $T\left(t\right)$, was normalized as shown in Equations (2), (3a) and (3b), and fitted to the single exponential function:

(4) $$\tilde{T}\left(t\right)\approx A\text{exp}\left(-\mathrm{Bt}\right)$$

by the least squares method with the Levenberg–Marquardt algorithm.

Full derivations of Equations (3a) and (3b) are given in Supporting Information (SI). It is noted that Equations (2), (3a) and (3b) refer to the volume averaged forms of solution to the HDE and that the fitted values of *A* are usually less than unity because of the truncation of the λ_n_ expansion (the details are found in SI).

Equating the value of *A* in Equation (4) with the prefactor in Equation (2), we can obtain the value of $\lambda_{1}$ as the minimum positive root for

(5) $$6\frac{1}{{\lambda_{1}}^{3}}\frac{{\left(\sin\left(\lambda_{1}\right)-\lambda_{1}\cos\left(\lambda_{1}\right)\right)}^{2}}{\left(\lambda_{1}-\sin\left(\lambda_{1}\right)\cos\left(\lambda_{1}\right)\right)}-A=0$$

for the spherically symmetric protein. As for Equations (3a) and (3b), we can obtain the value of $\lambda_{1}$ as the minimum positive root for

(6) $$4\frac{1}{{\lambda_{1}}^{2}}\frac{J_{1}{\left(\lambda_{1}\right)}^{2}}{J_{0}{\left(\lambda_{1}\right)}^{2}+J_{1}{\left(\lambda_{1}\right)}^{2}}-A=0$$

and

(7) $$\frac{4\cdot \sin^{2}\left(\lambda_{1}\right)}{\lambda_{1}\cdot \left(2\lambda_{1}+\sin\left(2\lambda_{1}\right)\right)}-A=0$$

respectively, for the radial and longitudinal directions of the cylindrically symmetric protein.

Then, using the estimated value of $B={\lambda_{1}}^{2}/\tau$, the thermal conductivity *κ* was calculated via:

(8) $$\kappa =\frac{l^{2}}{\tau }\frac{C_{p}}{V}$$

Here, *C*_*p*_ denotes the heat capacity of the protein. *V* refers to the volume ($\frac{4}{3}\pi {R_{s}}^{3}$) for sphere with radius of *R*_*s*_ or that ($\pi {R_{c}}^{2}H_{c}$) for cylinder with radius of *R*_*c*_ and height of *H*_*c*_. *l* represents the radius of sphere (*R*_*s*_), radius of cylinder (*R*_*c*_), or a half of height of cylinder (*H*_*c*_/2). In computing thermal conductivity and thermal conductance of protein, we took over the value of *C*_*p*_ from the previous study by Lervik et al.^31^ as *C*_*p*_ = 27 kJ/mol/K for Mb and *C*_*p*_ = 47 kJ/mol/K for GFP.

Lastly, the thermal conductance *G* at the protein‐water interface was obtained by using *l*, *κ* and $\lambda_{1}$ through (see SI)

(9) $$1-\lambda_{1}\cot\left(\lambda_{1}\right)=\frac{GR_{s}}{\kappa }=\mathrm{Bi}$$

(10) $$\frac{\lambda_{1}J_{1}\left(\lambda_{1}\right)}{J_{0}\left(\lambda_{1}\right)}=\frac{G_{//}R_{c}}{\kappa_{//}}=Bi_{//}$$

(11) $$\lambda_{1}\tan\left(\lambda_{1}\right)=\frac{G_{⊥}}{\kappa_{⊥}}\frac{H_{c}}{2}=Bi_{⊥}$$

Here, Equations (9), (10) and (11) correspond to Equations (2), (3a) and (3b), respectively. $\lambda_{1}$ is the minimum positive root of either Equations 5, 6 or 7. *Bi* denotes the Biot number which is given by *κ*, *G* and *l* (= *R*_*s*_, *R*_*c*_ or *H*_*c*_/2) for each system.

In estimating each of value sets of thermal conductivity and thermal conductance (Tables 2 and 3 below), we identified outliers by using the double‐side Smirnov‐Grubbs test with *p* value of 0.05 and ignored such outliers in the following statistical analyses.

**TABLE 2:** Evaluations of thermal conductivity *κ* and thermal conductance *G* of Mb and GFP in water for the spherical model and the radial (r) and longitudinal (z) directions of the cylindrical model obtained by averaging the calculated values for the box sizes of 3.0, 4.0 and 4.5 nm (see Figures 3 and 4)

	Gyration radius like size			Filling corrected size		
	Sphere	Cylinder (r)	Cylinder (z)	Sphere	Cylinder (r)	Cylinder (z)
κ for Mb	0.10 ± 0.02	0.18 ± 0.03	0.28 ± 0.08	0.08 ± 0.01	0.10 ± 0.02	0.24 ± 0.07
κ for GFP	0.15 ± 0.03	0.22 ± 0.04	0.86 ± 0.16	0.12 ± 0.02	0.13 ± 0.02	0.75 ± 0.14
G for Mb	255 ± 8	732 ± 90	2230 ± 840	151 ± 5	301 ± 37	1120 ± 420
G for GFP	309 ± 65	967 ± 265	3670 ± 670	185 ± 39	398 ± 109	1850 ± 340

*Note*: Two types of evaluations obtained with the gyration radius like and the filling corrected estimations for protein size are shown. *κ* and G are measured in units of W/K/m and MW/K/m^2^, respectively.

**TABLE 3:** Evaluations of thermal conductivity *κ* and thermal conductance *G* of Mb and GFP in 140 mM NaCl aqueous solution for the spherical model and the radial (r) and longitudinal (z) directions of the cylindrical model obtained by averaging the calculated values for the box sizes of 3.0, 4.0 and 4.5 nm (see Figures 7 and 8)

	Gyration radius like size			Filling corrected size		
	Sphere	Cylinder (r)	Cylinder (z)	Sphere	Cylinder (r)	Cylinder (z)
κ for Mb	0.11 ± 0.02	0.19 ± 0.03	0.28 ± 0.05	0.08 ± 0.01	0.11 ± 0.02	0.25 ± 0.05
κ for GFP	0.17 ± 0.08	0.25 ± 0.12	0.97 ± 0.49	0.13 ± 0.06	0.14 ± 0.07	0.84 ± 0.43
G for Mb	234 ± 30	671 ± 154	2300 ± 810	138 ± 18	276 ± 63	1160 ± 410
G for GFP	316 ± 28	1009 ± 70	4250 ± 810	190 ± 17	416 ± 29	2140 ± 410

*Note*: Two types of evaluations obtained with the gyration radius like and the filling corrected estimations for protein size are shown. *κ* and G are measured in units of W/K/m and MW/K/m^2^, respectively.

## 3 RESULTS AND DISCUSSION

### 3.1 Spherically symmetric cases

First, we consider the spherical model for protein, where the radius of protein is evaluated as the gyration radius (see SI) in accord with the earlier study by Lervik et al.^31^ With the use of various sizes of rectangular box (whose distance from protein surface to box boundary ranges between 1.0 and 4.5 nm) under the periodic boundary condition, in which a protein is surrounded by solvating water molecules, we calculated the temporal evolution of spatially averaged protein temperature *T*(*t*) so that the thermal conductivity *κ* and the thermal conductance *G* were evaluated. An example for the behavior of *T*(*t*) is shown in Figure 2 for the case of Mb in the water box of 1 nm size.

In contrast to the previous study^31^ in which the water sphere model was employed for the solvation, the present study relied on the periodic boundary condition as mentioned above. While the water sphere model may be efficient for spherical (or globular) protein, it would be inefficient for non‐spherical protein in order to keep the sufficient numbers of solvating water molecules. It is then expected that the use of the rectangular water box under the periodic boundary condition could efficiently reduce the number of water molecules surrounding the non‐spherical protein, thus enabling the accurate non‐equilibrium temperature relaxation simulation for solvated protein with reasonable computational cost in general.

**FIGURE 3:**
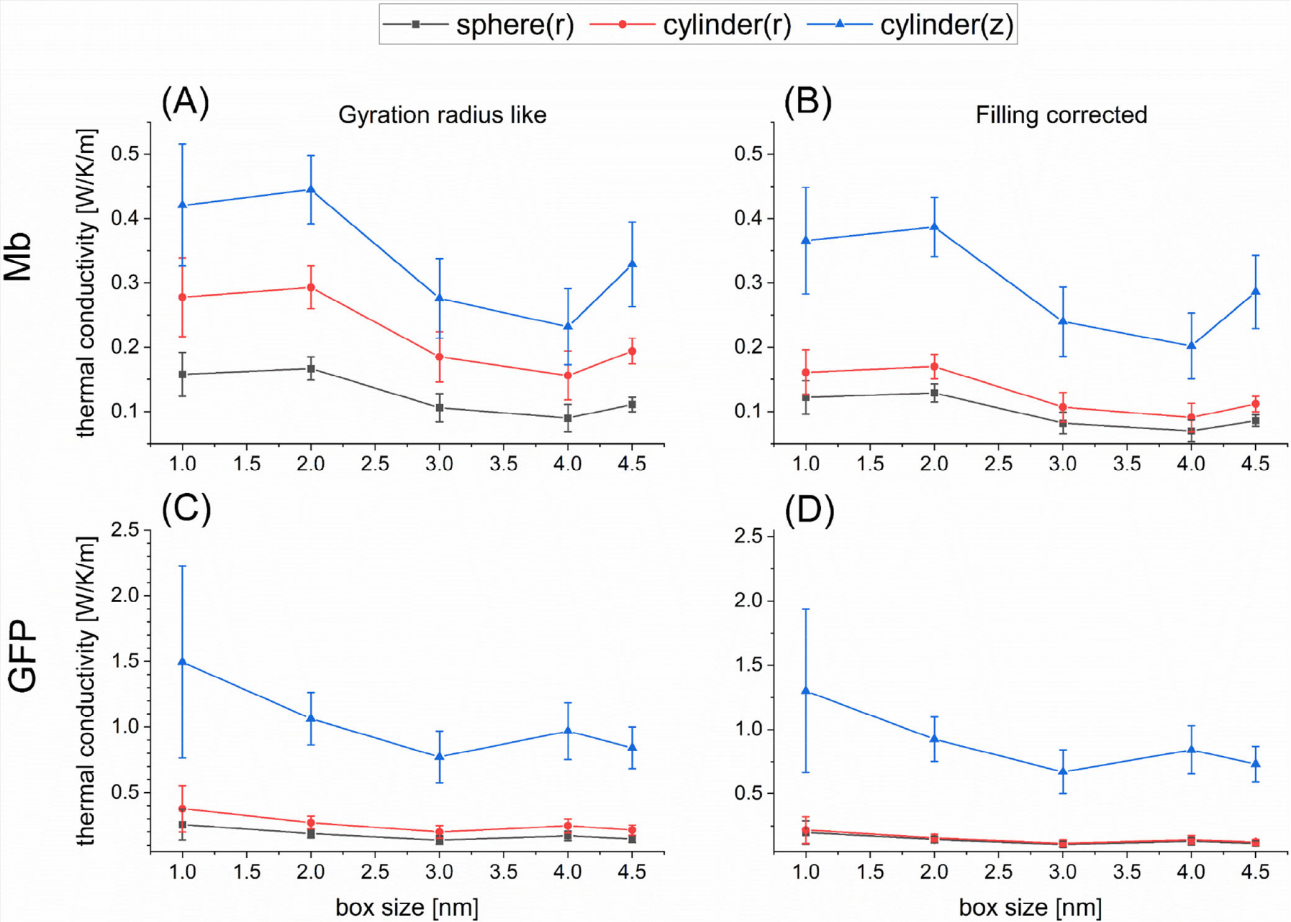
Evaluations of thermal conductivity *κ* of protein in water for the spherical model (black squares) and the radial (red circles) and longitudinal (blue triangles) directions of the cylindrical model as functions of simulation box size (1.0–4.5 nm). (A) Results for Mb obtained with the gyration radius like estimation for protein size; (B) results for Mb obtained with the filling corrected estimation for protein size; (C) results for GFP obtained with the gyration radius like estimation for protein size; (D) results for GFP obtained with the filling corrected estimation for protein size.

**FIGURE 4:**
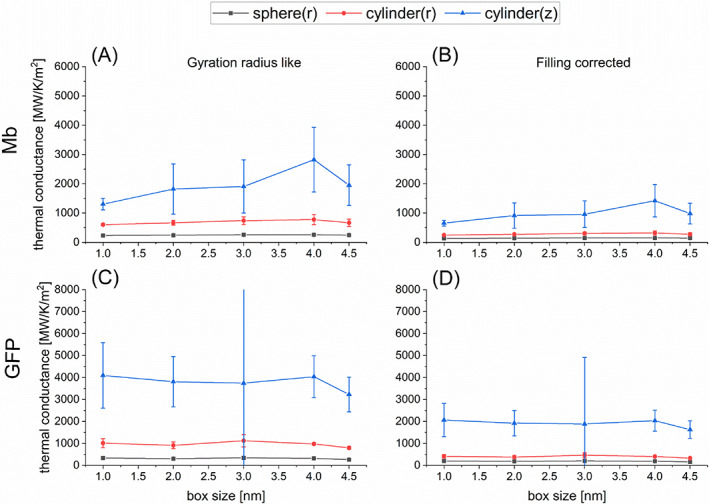
Evaluations of thermal conductance *G* of protein in water for the spherical model (black squares) and the radial (red circles) and longitudinal (blue triangles) directions of the cylindrical model as functions of simulation box size (1.0–4.5 nm). (A) Results for Mb obtained with the gyration radius like estimation for protein size; (B) results for Mb obtained with the filling corrected estimation for protein size; (C) results for GFP obtained with the gyration radius like estimation for protein size; (D) results for GFP obtained with the filling corrected estimation for protein size.

The calculated values of *κ* and *G* for Mb and GFP are illustrated in Figures 3 and 4 for five box sizes. We thus observe that the calculated values almost converge when the box size reaches 3 nm. These estimated values of *κ* and *G* averaged using the calculated data for the box sizes being 3.0, 4.0 and 4.5 nm are summarized in Table 2. We thus find that the present evaluations of *κ* for Mb and GFP are similar to those in the earlier studies^20^, ^30^, ^31^, ^33^ (for example,^31^ 0.13 ± 0.02 W/K/m for Mb and 0.12 ± 0.01 W/K/m for GFP), while the evaluations for *G* somewhat deviate from the earlier ones^31^, ^34^, ^46^, ^47^ (for example,^46^, ^47^ 301 MW/K/m^2^ for Mb and 329 MW/K/m^2^ for GFP at 300 K). The reason for the latter (insignificant) discrepancy may be attributed to the delicate dependency of parameter fitting on the behavior of *T*(*t*) (see also the following section). It is also remarked that the calculated values of *κ* for Mb and GFP are much lower than the thermal conductivity of pure water, which may be evaluated^48^ to be higher than 0.6 W/K/m. This result may make sense when we attempt to reconcile the gap issue^12^, ^13^, ^14^, ^15^ between the experimentally observed temperature inhomogeneity in living cells and the theoretical expectation using the thermal conductivity of water.

Here, we pay attention to a fact that the evaluated values of *κ* and *G* sensitively depend on the estimated protein size, as revealed in Equations (8) and (9). In the above analysis, which is analogous to the previous study,^31^ the effective radius of spherical protein has been evaluated as the gyration radius (that is, 1.529 nm for Mb and 1.667 nm for GFP according to Lervik et al.^31^). However, if a protein is regarded as a homogeneous sphere, its real radius is larger than the gyration radius by the factor of (5/3)^1/2^ = 1.29 (see SI‐1). In addition, in light of the physical meaning of thermal conductance defined through the temperature gap at the protein‐water interface, it would be desirable to employ the real radius above instead of the gyration radius. We considered this issue and increased the protein radius by this factor (5/3)^1/2^ compared to the gyration radius for the evaluation of thermal conductivity and conductance. Thus, the estimated values of *κ* and *G* were reduced by 0.77 and 0.60, respectively. These estimated values are illustrated in Figures 3 and 4 and Table 2 as well. We suggest the use of the “actual” radius multiplied by (5/3)^1/2^ to the gyration radius for more reliable evaluation of *κ* and *G*, which is referred to as “filling corrected” radius in this work.

As a final remark in this subsection, the averaged values of Biot number (*Bi* in Equation [9]) were estimated for Mb and GFP. In each of simulation systems, the *Bi* value is significantly larger than unity (4.0 ± 0.7 for Mb and 4.6 ± 1.9 for GFP), indicating that the heat conduction inside protein molecule progresses relatively slowly compared to that in surrounding solvent (water in this case). It may thus be remarked that we could not ignore the appearance of temperature gradient inside the solvated proteins such as Mb and GFP.

### 3.2 Cylindrically symmetric cases

In the preceding section, we have regarded a protein as a spherical body. However, the shape of protein is often non‐spherical; for example, GFP seems to be better approximated by a cylindrical body (see Figure 1). Therefore, we consider in the following the thermal conductivity and conductance for the protein with cylindrical symmetry (see also SI).

First, we discuss the size estimation of cylindrical protein and its relationship to the evaluation of thermal conductivity and conductance. Let us consider a uniform cylinder with the radius *R*_*c*_ and the height *H*_*c*_ whose volume is π*R*_*c*_^2^ *H*_*c*_. As shown in SI‐1, the radii of gyration extended to the cylindrically symmetric case, which are evaluated through the variances of location around the center of mass, are $r_{c}=\frac{R_{c}}{\sqrt{2}}$ and $\frac{h_{c}}{2}=\frac{H_{c}}{2\sqrt{3}}$ for radial and vertical directions, respectively. Therefore, as has been illustrated for a spherical protein above, if we evaluate the thermal conductivity and conductance for a cylindrical protein by employing the (extended) gyration radii to model the protein size, we would overestimate the values of *κ* and *G* compared with those evaluated with *R*_*c*_ and *H*_*c*_/2.

Next, we study the difference in the estimated size of protein between the two cases when a protein is regarded as a spherical or cylindrical body. Again, let us consider a uniform cylinder with the radius *R*_*c*_ and the height *H*_*c*_ whose volume is π*R*_*c*_^2^ *H*_*c*_. As mentioned above, when we consider the cylindrical body, the radii of gyration are $r_{c}=\frac{R_{c}}{\sqrt{2}}$ and $\frac{h_{c}}{2}=\frac{H_{c}}{2\sqrt{3}}$ for radial and vertical directions, respectively. On the other hand, if we evaluate a spherical radius of gyration *r*_*s*_ for this uniform cylinder by calculating the variances of location (*x* ^*2*^ *+ y* ^*2*^ *+ z* ^2^) from the center over the cylinder, we find $r_{s}^{2}=\frac{R_{c}^{2}}{2}+\frac{H_{c}^{2}}{12}$ (see SI‐1). Therefore, the volume ratio between the sphere with radius *r*_*s*_ and the cylinder with radius *r*_*c*_ and height *h*_*c*_ is

(12) $$\frac{{\tilde{V}}_{\text{radgy},s}}{V_{\text{radgy},c}}=\frac{8\sqrt{3}}{3}{\left(\frac{1}{2}{\left(\frac{R_{c}}{H_{c}}\right)}^{\frac{2}{3}}+\frac{1}{12}{\left(\frac{H_{c}}{R_{c}}\right)}^{\frac{4}{3}}\right)}^{\frac{3}{2}}$$

where ${\tilde{V}}_{\text{radgy},s}$ and *V*_*radgy,c*_ refer to the volumes of the spherical and cylindrical models, respectively. Figure S1 in SI illustrates this ratio as a function of *R*_*c*_ */H*_*c*_, thus showing that the spherical approximation to the cylindrical body overestimates the volume significantly, in particular when *R*_*c*_ */H*_*c*_ deviates from $1/\sqrt{3}$, where Equation (12) takes the minimum value $\sqrt{3}$. Consequently, the application of the spherical approximation to a cylindrical protein would substantially affect the evaluation of thermal conductivity and conductance (see SI‐1).

On the basis of the computational scheme shown in Section 2.2, we first evaluated the thermal conductivity *κ* and the thermal conductance *G* for Mb and GFP using the cylindrical model in which the gyration radius *r*_*c*_ and the (effective, gyration‐like) height *h*_*c*_ were evaluated with the method illustrated in SI. In addition, we calculated *κ* and *G* by using the radius $R_{c}=\sqrt{2}r_{c}$ and the height $H_{c}=\sqrt{3}h_{c}$ (“filling corrected” values) to correct the size of protein, as in the case of the spherical model. Figures 3 and 4 show the calculated values of *κ* and *G*, respectively, as functions of box size, where the values obtained with (*r*_*c*_, *h*_*c*_) and (*R*_*c*_, *H*_*c*_) are compared. The estimated values of *κ* and *G* are summarized in Table 2, where averaging over the calculated values for the box sizes of 3.0–4.5 nm was carried out.

As seen in these results, the estimated values of *κ* in the cylindrical model are higher than those in the spherical model both for the radial (*r* or //) and vertical (*z* or ⊥) directions. In the comparison between the radial and vertical directions, *κ*_⊥_ is significantly higher than *κ*_//_. For example, we find *κ*_⊥_ = 0.86 W/K/m and *κ*_//_=0.22 W/K/m compared to *κ* = 0.15 W/K/m for the spherical model in the case of GFP employing the gyration‐radius‐like size estimation. The evaluations obtained with (*R*_*c*_, *H*_*c*_) give lower values of *κ* compared to those with (*r*_*c*_, *h*_*c*_), that is, by factor of 0.58 for *κ*_//_ and factor of 0.87 for *κ*_⊥_. These results thus suggest an importance of carefully accounting for the geometrical shape of protein. In particular, the present analysis shows that a remarked enhancement of thermal conduction may be expected along the longitudinal direction of protein with cylindrical shape. Concerning the evaluations for *G*, we also observe the higher values in the cylindrical model than those in the spherical model. The thermal conductance for the longitudinal direction is much higher than that for the radial direction in the cylindrical model, and their values are reduced by the factors of 0.41 and 0.50 in the radial and longitudinal directions, respectively, by employing the protein size of (*R*_*c*_, *H*_*c*_) instead of (*r*_*c*_, *h*_*c*_). However, these results should not be taken literally but should be considered with caution. The reasons are explained in the following.

**FIGURE 5:**
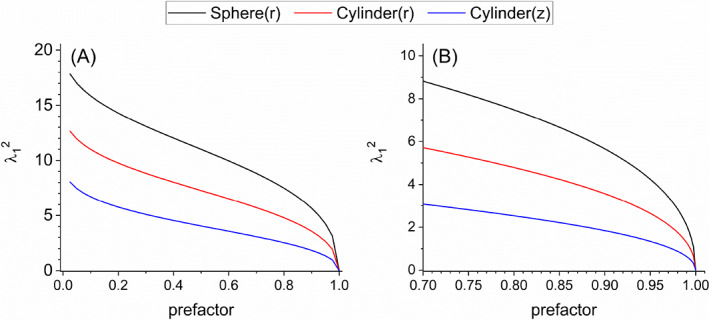
Relationships between the prefactor *A* in Equation (4) and the values of *λ*_1_^2^ as the solutions *λ*_1_ to Equations ((5), (6), (7)) for the spherical and cylindrical (*r* and *z* directions) models, respectively. Left: Relational curves (sphere: black, cylinder (*r*): red, cylinder (*z*): blue) for 0 < *A* < 1. Right: Zoomed range for 0.7 < *A* < 1.0.

**FIGURE 6:**
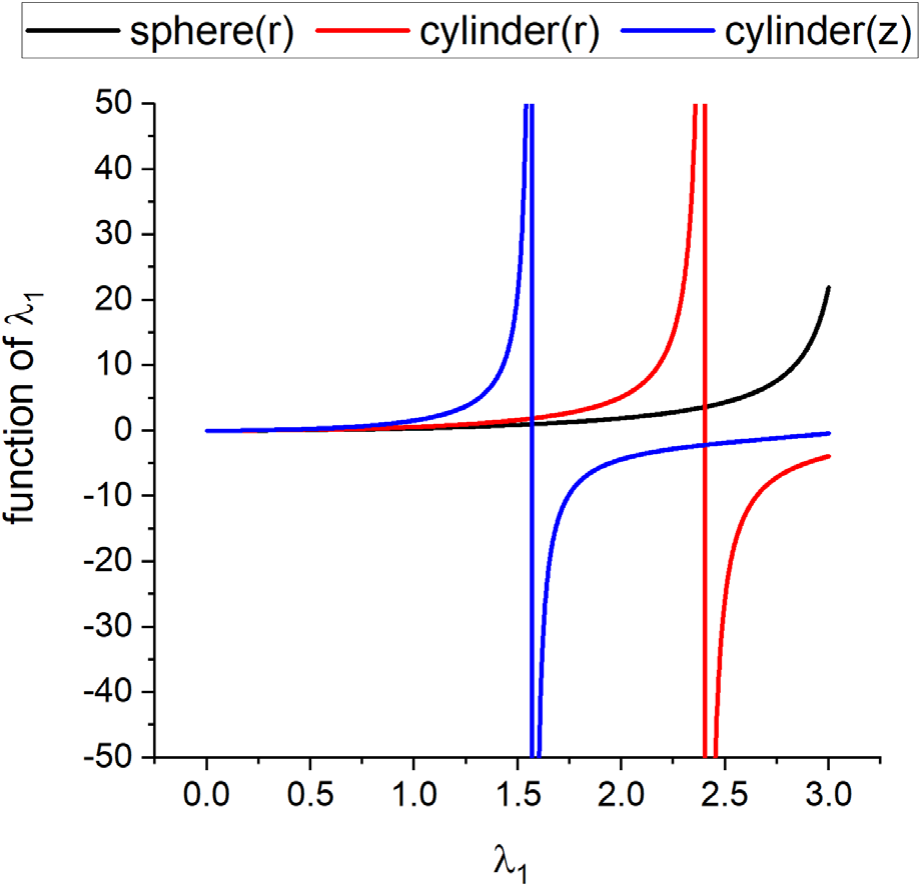
Functions of *λ*_1_ that appear on the left‐hand sides of Equations ((9), (10), (11)). Black, red, and blue curves refer to the functional relations in the sphere, cylinder (*r*) and cylinder (*z*) models, respectively.

As seen in Section 2.2, the parameters λ_1_ and τ are estimated through the fitting for the time dependence *T*(*t*) of spatially averaged temperature of protein. These estimated parameters then determine *κ* and *G* through Equations ((8), (9), (10), (11)). Thus, we see the evaluations of the thermal conductivity and conductance are sensitive to the values of λ_1_ which are related to the prefactor *A* as shown in Equations ((5), (6), (7)) and Figure 5. Concerning the estimation of thermal conductance *G*, the left‐hand sides of Equations ((9), (10), (11)) as functions of λ_1_ are also crucial, which are highly dependent on λ_1_ as seen in Figure 6. In this way, the present computational approach to the evaluations of *κ* and *G* is somewhat vulnerable regarding the stability of solutions, thus demanding the careful interpretation of the calculated results. In particular, we have observed that the evaluations of *G* for the longitudinal direction in the cylindrical model are very sensitive to the estimation of λ_1_, possibly causing somewhat enhanced estimations. We may also remark a possibility that retaining only the first term of the λ_n_ expansion (see SI) would cause some inaccuracies depending on the directions of thermal conduction.

In addition, we have evaluated *κ* and *G* individually for the radial and longitudinal directions in the cylindrical model. As seen in SI‐5, it is possible in principle that we could analyze the thermal conduction both for the radial and longitudinal directions simultaneously on the basis of Equation 59 in SI, whereas it was practically infeasible in the present scheme. When we would explain the temporal change of protein temperature only in terms of the heat flux of either radial or longitudinal direction, we would overestimate the values of thermal conductivity and conductance. For example, if we assume that the radial and longitudinal contributions to the total heat flux are proportionally given as *p*: *q*, respectively, we would overestimate the former and latter contributions by the factors of (*p + q*)/*p* and (*p + q*)/*q*, respectively, when only one contribution is considered. Therefore, it is reasonable to correct the values of *κ* and *G* by *p*/(*p + q*) and *q*/(*p + q*) for the radial and longitudinal directions, respectively, in this modeling. Although the accurate estimation of the ratio of *p: q* is somewhat difficult, we may roughly evaluate it in proportion to the cross section of heat flux for the both directions. When we consider a cylinder with the radius *R* and the height *H*, the ratio of the two cross sections is *2πRH: 2πR* ^*2*^ *= H: R*, which may approximate the ratio *p: q*. This correction could fairly suppress the calculated values of *κ* and *G* in the cylindrical model.

Furthermore, we have constrained the motion of hydrogen atoms by means of the LINCS algorithm in our MD simulation. Though this modeling was also used in the earlier study^31^ to reduce the computational cost, the employed fixing of hydrogen motions may subtly reduce the estimated values of thermal conductivity. Performing the higher‐cost simulations to consider the flexible hydrogen model is beyond the scope of the present study and would be a remaining problem in a future study.

Altogether, in spite of some uncertainties, through the consideration of anisotropy and the inclusion of the periodic boundary condition on the simulation cell, the present methodology can provide an efficient and accurate tool for the estimation of the thermal conductivity and conductance of solvated proteins with more variable geometrical shapes so that the computed values of *κ* are comparable to experimental values; for example, the *κ* values for Mb shown in Table 2 may be compared to an experimental value of *κ* = 0.194 ± 0.024 W/K/m.^20^ As for the *G* values, we have no experimental estimation available for the comparison to the present calculated results. We may then compare the present results to some theoretical evaluations,^31^, ^34^, ^46^, ^47^ thus finding reasonable consistencies.

### 3.3 Effects of salt addition to water solvent

**FIGURE 7:**
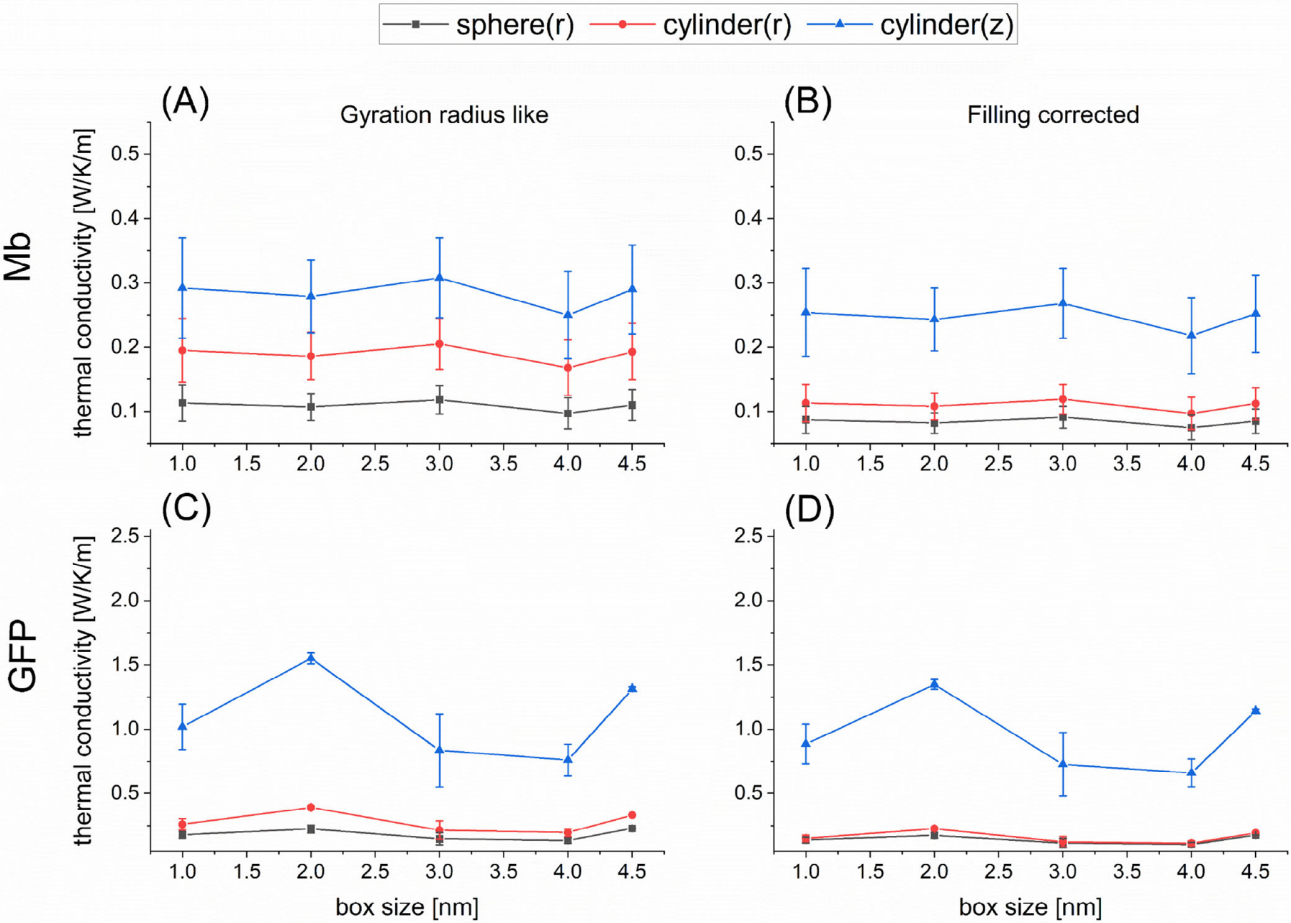
Evaluations of thermal conductivity *κ* of protein in 140 mM NaCl aqueous solution for the spherical model (black squares) and the radial (red circles) and longitudinal (blue triangles) directions of the cylindrical model as functions of simulation box size (1.0–4.5 nm). (A) Results for Mb obtained with the gyration radius like estimation for protein size; (B) results for Mb obtained with the filling corrected estimation for protein size; (C) results for GFP obtained with the gyration radius like estimation for protein size; (D) results for GFP obtained with the filling corrected estimation for protein size.

**FIGURE 8:**
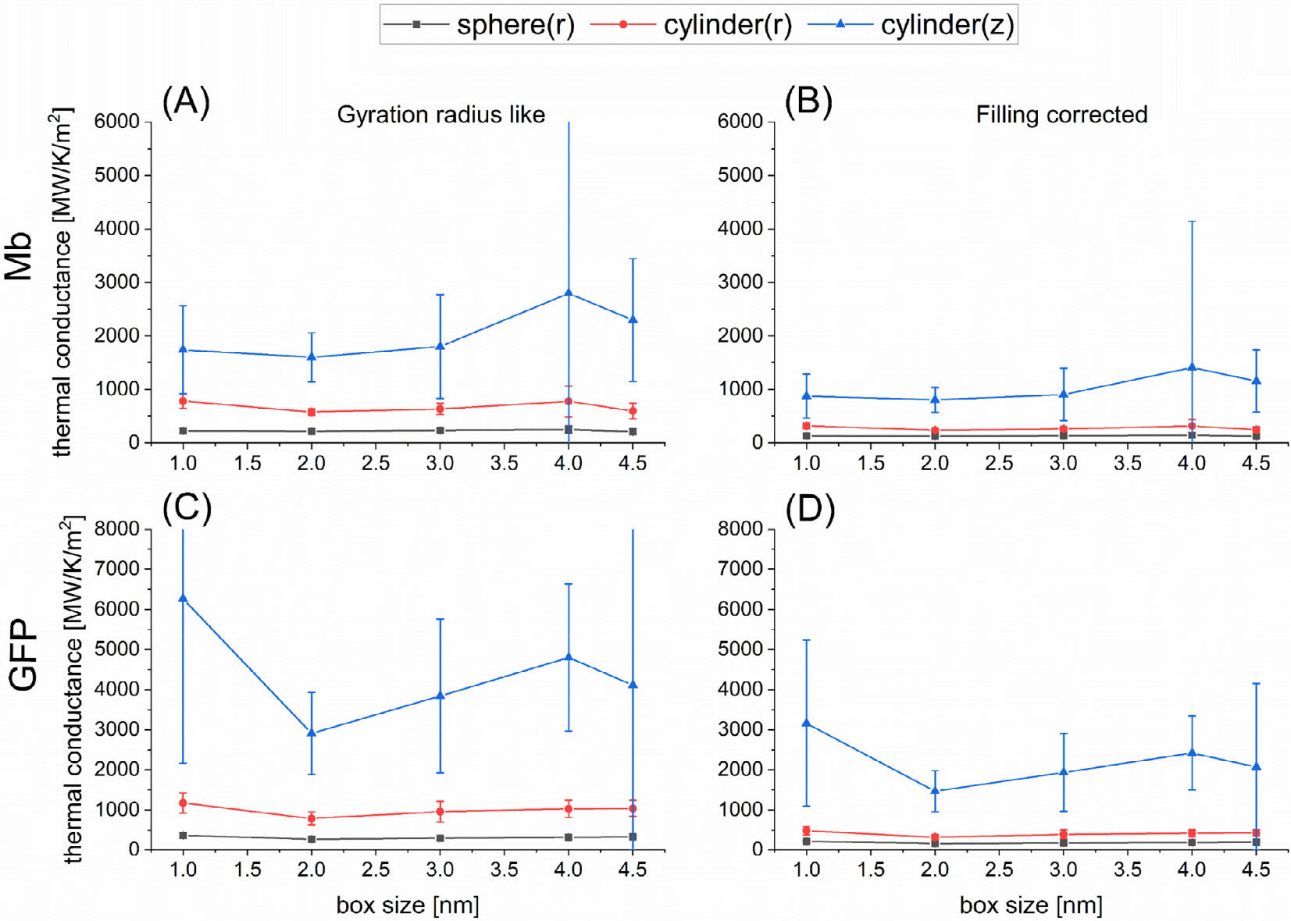
Evaluations of thermal conductance *G* of protein in 140 mM NaCl aqueous solution for the spherical model (black squares) and the radial (red circles) and longitudinal (blue triangles) directions of the cylindrical model as functions of simulation box size (1.0–4.5 nm). (A) Results for Mb obtained with the gyration radius like estimation for protein size; (B) results for Mb obtained with the filling corrected estimation for protein size; (C) results for GFP obtained with the gyration radius like estimation for protein size; (D) results for GFP obtained with the filling corrected estimation for protein size.

Molecular properties of proteins (e.g., thermal stability) are significantly affected by interaction with co‐solute molecules such as ions and metabolites solved in the aqueous solution. Thus, supposing the atomistic environmental effects on the thermal conductivity and conductance of protein, we next calculated *κ* and *G* for Mb and GFP in the presence of Na and Cl ions with the concentration of 140 mM in the aqueous solution. Figures 7 and 8 illustrate the calculated results of *κ* and *G*, respectively, for Mb and GFP as the functions of box size, where both the results obtained with the gyration radii and the corrected size for protein (see above) are shown. The statistically averaged evaluations thereof using the values for the box sizes of 3.0, 4.0 and 4.5 nm are then given in Table 3. Overall, the effects of salt addition with 140 mM NaCl, that is a physiologically relevant concentration in living cells, are fairly minor concerning the changes in the values of *κ* and *G*. (Both the results with and without the salt addition agree with each other within the statistical errors.) This finding is consistent with the insights obtained in the previous investigations^46^, ^47^ based on the diffuse mismatch model, in which the thermal conductance was evaluated through the vibrational density of states of protein and surrounding medium; unless the addition of salt substantially affects the density of states of aqueous solution, one would not expect a significant impact on the thermal conductance. Then, further simulations employing other crowding molecules such as saccharides, glycans, polyethylene glycol (PEG), nucleic acids and other proteins would be interesting as future studies. For example, Pandey and Leitner^34^ computationally investigated the thermal transport between protein and water through a trehalose layer, and found a significant reduction in thermal conductance by several times. Such a “blanket” effect by saccharides makes a marked contrast to the present result for salt addition and its mechanism remains to be elucidated in more details.

## 4 CONCLUSION

In this study we have proposed a computational scheme to evaluate the thermal conductivity *κ* and conductance *G* of protein in aqueous solution, where the methodology^31^ based on the all‐atom non‐equilibrium MD simulation was extended so as to account for the cylindrical shape of protein in addition to the spherical shape under the periodic boundary condition for the hydrated system. We have thus calculated the values of *κ* and *G* for Mb and GFP both for the spherical and cylindrical models for protein, where the values for the radial and longitudinal directions were estimated individually in the latter model. Also considering the correction for the size estimation of protein that was treated in terms of the gyration radius in the earlier study,^31^ the present study has given the quantitative descriptions of thermal conduction in solvated protein that are consistent with earlier computational and experimental data as a whole. Meanwhile, we have found a possibility that the thermal conduction along the longitudinal (*z*) direction in the cylindrical model may be enhanced substantially, though this result is sensitively dependent on the geometrical shape of protein and also on the approximations employed for the parameter (in particular, λ_1_) evaluation and the separate treatment of thermal conductions along the two directions. We have also found that the addition of 140 mM (physiological concentration) NaCl to solution does not significantly modify the estimated values of *κ* and *G*. Further studies on these issues for the anisotropic thermal conduction (also including the case for membrane proteins such as G protein‐coupled receptors) and the co‐solvent effects are expected to be done in the future.

## Supporting information


**Data S1:** Supporting Information.

## Data Availability

The data that support the findings of this study are available from the corresponding author upon reasonable request.
