# Supplementary material for: Thermal conductivity and conductance of protein in aqueous solution: Effects of geometrical shape
Source: J Comput Chem. 2022 Dec 5;44(7):857–68. doi: 10.1002/jcc.27048 (PMC10107505; doi:10.1002/jcc.27048)
Supplement: Supplementary file 1 — Data S1: Supporting Information. [file JCC-44-857-s001.pdf]

Supporting Information for

Thermal conductivity and conductance of protein in aqueous solution: Effects of geometrical shape

Ikuo Kurisaki[1], Seiya Tanaka[2], Ichiro Mori[2], Toshihito Umegaki[1,3], Yoshiharu Mori[1], Shigenori Tanaka[1]

[1]Graduate School of System Informatics, Kobe University, 1-1 Rokkodai, Nada, Kobe 657-8501, Japan

[2]Graduate School of Science, Technology and Innovation, Kobe University, 1-1 Rokkodai, Nada, Kobe 657-8501, Japan

[3]Center for Mathematical Modeling and Data Science, Osaka University, Osaka, 560-8531, Japan

SI-1 Gyration-radius-like geometry measure for uniform sphere and cylinder

Lervik and colleagues[1] used a radius of gyration for a measure of spherical protein size, whereas their idea to describe protein size is not unique. Here, we consider an ideal sphere or cylinder with uniform mass and compare the radius of gyration (or gyration radius-like geometry measure) with the given geometry measure.

First, we discuss a sphere of uniform mass with radius of R_s, where the center of mass is located at the origin. The mass density (total mass is taken to be unity) and volume are given as follows:

$$\rho(x,y,z) = \begin{cases} \frac{1}{V_{exact,s}} \left(if\ 0 \le x^2+y^2+z^2 \le {R_s}^2\right) \\ 0\ (else) \end{cases} \qquad (1)$$

$$V_{exact,s} = \frac{4\pi}{3}{R_s}^3 \qquad (2)$$

Then, we can analytically calculate the radius of gyration, $r_{s,radgy}$:

$${r_{s,radgy}}^2 = \int\int\int_{0\le x^2+y^2+z^2\le R^2} dxdydz \cdot (x^2+y^2+z^2)\rho(x,y,z)$$
$$= \int_0^R dr \int_0^\pi d\theta \int_0^{2\pi} d\varphi \cdot r^2\rho(x,y,z) \cdot r^2 \sin\theta$$
$$= \frac{3}{5}{R_s}^2 \qquad (3)$$

We can thus find that the given radius, R_s, is $\sqrt{\frac{5}{3}} \approx 1.29$-fold larger than the radius of gyration. 1.29 can be a scale factor to correct the value of $r_{s,radgy}$ for the actual radius value. Recalling eqn. 8 in the main text, using $r_{s,radgy}$ for estimation of κ leads to the

overestimation by 1.29.

Second, we consider a cylinder of uniform mass with radius of R_c and height of H_c, where the center of mass is located at the origin. The mass density and volume are given as follows:

$$\rho(x,y,z) = \begin{cases} \frac{1}{V_{exact,c}} \left(if\ 0 \le x^2 + y^2 \le {R_c}^2; -\frac{H_c}{2} \le z \le \frac{H_c}{2}\right) \\ 0\ (else) \end{cases} \quad (4)$$

$$V_{exact,c} = \pi {R_c}^2 H_c \quad (5)$$

Then, we can analytically calculate the radii of gyration extended for the cylindrical radius and height, denoted as $r_{c,radgy}$ and $h_{c,radgy}$:

$$\begin{aligned} {r_{c,radgy}}^2 &= \int\int_{0\le x^2+y^2\le {R_c}^2} dxdy \int_{-\frac{H_c}{2}}^{\frac{H_c}{2}} dz \cdot (x^2+y^2)\rho(x,y,z) \\ &= \int_0^{R_c} dr \int_0^{2\pi} d\theta \int_0^{H_c} dz \cdot r \cdot r^2 \rho(x,y,z) \\ &= \frac{1}{2}{R_c}^2 \quad (6) \end{aligned}$$

$$\left(\frac{h_{c,radgy}}{2}\right)^2 = \int\int_{0\le x^2+y^2\le {R_c}^2} dxdy \int_{-\frac{H_c}{2}}^{\frac{H_c}{2}} dz \cdot z^2 \rho(x,y,z) = \frac{1}{12}{H_c}^2 \quad (7)$$

Accordingly, correction factors to recover the given geometry measures are c.a. $\sqrt{2} \approx 1.41$ and $\sqrt{3} \approx 1.73$ for the radius and height, respectively. Furthermore, we can calculate an effective gyration radius of the cylinder regarded as a sphere, ${r^{s|c}}_{radgy}$; the formula is given as:

$$\left({r^{s|c}}_{radgy}\right)^2 = \int\int_{0\le x^2+y^2\le {R_c}^2} dxdy \int_{-\frac{H_c}{2}}^{\frac{H_c}{2}} dz \cdot (x^2+y^2+z^2)\rho(x,y,z)$$

$$= \int_0^{R_c} dr \int_0^{2\pi} d\theta \int_{-\frac{H_c}{2}}^{\frac{H_c}{2}} dz \cdot r\,(r^2 + z^2)\rho(x, y, z)$$

$$= \frac{1}{2}{R_c}^2 + \frac{1}{12}{H_c}^2 = {r_{radgy}}^2 + \frac{1}{4}{\hbar_{radgy}}^2 \quad (8)$$

$${r^{s|c}}_{radgy} = \sqrt{{r_{radgy}}^2 + \frac{1}{4}{\hbar_{radgy}}^2} \quad (9)$$

According to eqn. 8 in the main text, the thermal conductivity is proportional to $\frac{l^2}{V}$, so that a given length measure l and a calculated protein volume V are important factors to determine the value of thermal conductivity, κ. It is thus worthwhile comparing the values of κ between different geometry definitions.

First, we consider the effect of correction of geometrical measure (tentatively given by the gyration radius) for the sphere with radius of R_s. As shown above, $r_{s,radgy}$ is c.a. 0.77-fold smaller than R_s. Using $r_{s,radgy}$ as an effective radius, the volume becomes less than half of the exact volume of the sphere as shown below:

$$V_{s,radgy} = \frac{4\pi}{3}\left(r_{s,radgy}\right)^3 = \left(\frac{3}{5}\right)^{\frac{3}{2}} \frac{4\pi}{3} {R_s}^3 \approx 0.465 \cdot V_{exact,s} \quad (10)$$

This gives two cautions to use the radius of gyration to describe the shapes of proteins. One is that the value of κ is overestimated by 1.29-folds. The other is that the boundary between a protein and the solution environment is not correctly described due to the smaller radius value defined as the radius of gyration. From theoretical point of view, it seems to be reasonable to numerically correct $r_{s,radgy}$, which is calculated from the atomic coordinates of protein, by multiplying the factor of 1/0.77 = 1.29.

Second, we consider correction effects for the cylinder with radius of R_c and height of H_c. As addressed above, $r_{c,radgy}$ and $h_{c,radgy}$ are 0.71-fold and 0.58-fold smaller than R_c and H_c, respectively. As for the volume calculated by using these gyration-like measures, we find

$$V_{radgy,c} = \pi {r_c}^2 h_c = \pi \left(\frac{R_c}{\sqrt{2}}\right)^2 \left(\frac{H_c}{\sqrt{3}}\right) \approx 0.29 \cdot V_{exact,c} \qquad (11)$$

Thus, using the radius and height of gyration-like geometry measure leads to c.a. 1.73-fold (radial direction) and 1.15-fold (longitudinal direction) larger values of κ than those for the cylinder with the actual radius and height values, R_c and H_c, respectively.

Besides, it is also interesting to consider the effect of the choice of geometrical model, either a sphere or cylinder. If we use the effective sphere radius ${r^{s|c}}_{radgy}$ for the cylindrical body with the radius R_c and the height H_c, the effective spherical volume can be calculated as $\frac{4}{3}\pi\left({r^{s|c}}_{radgy}\right)^3$, referred to as $\tilde{V}_{radgy,s}$. The ratio of $\tilde{V}_{radgy,s}$ to $V_{radgy,c}$ is then given by

$$\frac{\tilde{V}_{radgy,s}}{V_{radgy,c}} = \frac{8\sqrt{3}}{3}\left(\frac{1}{2}\left(\frac{R_c}{H_c}\right)^{\frac{2}{3}} + \frac{1}{12}\left(\frac{H_c}{R_c}\right)^{\frac{4}{3}}\right)^{\frac{3}{2}} \qquad (12)$$

As illustrated in Figure S1, this ratio is greater than unity, indicating that the sphere model overestimates the volume of protein with cylindrical shape.

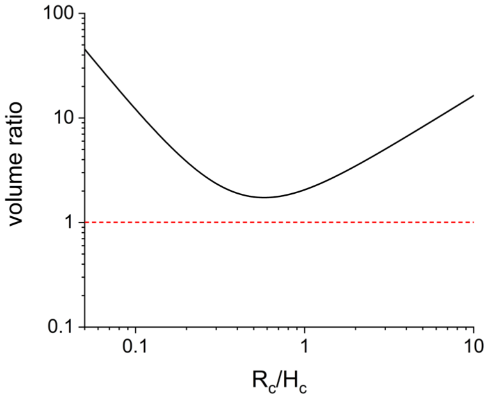

Figure S1. Volume ratio of sphere model (with gyration radius $r^{s|c}_{radgy}$) to cylinder model (with gyration radius $r_{c,radgy}$ and gyration height $h_{c,radgy}$) for uniform cylinder as a function of R_c/H_c, where R_c and H_c denote the actual radius and height of cylinder, respectively.

SI-2 Calculation of geometry measure (radius and height) for cylinder-like body by using atomic coordinates of protein molecule

We calculate the radius and height values for the two model proteins, myoglobin (Mb) and green fluorescent protein (GFP), described in terms of the cylindrical model as follows. First, we choose two Cα carbon atoms, which appear to be relatively stable in the protein (such as those located in an α-helix), and define a unit vector, $\vec{n}_a = \frac{\vec{x}_2 - \vec{x}_1}{|\vec{x}_2 - \vec{x}_1|} = \left(n_x, n_y, n_z\right)$. $\vec{x}_1$ and $\vec{x}_2$ are the position vectors of one and the other Cα carbon atoms, respectively. Then, using this vector, we obtain another unit vector vertical to $\vec{n}_a$, referred to as $\vec{n}_b$. The component of $\vec{n}_b$ is given by

$$\vec{n}_b = \frac{1}{\sqrt{{b_x}^2 + {b_y}^2 + \left(\frac{-n_x b_x - n_y b_y}{n_z}\right)^2}} \cdot \left(b_x, b_y, \frac{-n_x b_x - n_y b_y}{n_z}\right) \quad (13)$$

It is noted that the atomic coordinate, $\left(b_x, b_y, \frac{-n_x b_x - n_y b_y}{n_z}\right)$, is found on the plane which is vertical to $\vec{n}_a$. b_x and b_y can be selected arbitrarily and we assign 1.0 to both of them in this study. Finally, we can estimate geometry measures by projecting atomic coordinates of protein onto $\vec{n}_a$ and $\vec{n}_b$. Assuming that $\{X_i^a\}$ and $\{X_i^b\}$ are projected values for $\vec{n}_a$ and $\vec{n}_b$, the standard deviations

$$M_a = \sqrt{\frac{1}{N_a}\sum_{i=1}^{N_a}(X_i^a - X_{ave}^a)^2}\,;\, M_b = \sqrt{\frac{1}{N_b}\sum_{i=1}^{N_b}\left(X_i^b - X_{ave}^b\right)^2} \quad (14)$$

can be considered as geometry measures similar to the radius of gyration. N_a and N_b are the size of data for $\{X_i^a\}$ and $\{X_i^b\}$, respectively. Meanwhile X_{ave}^a and X_{ave}^b are the

average values of $\{X_i^a\}$ and $\{X_i^b\}$, respectively.

The radius of Mb is determined by defining $\vec{n}_a$ as a vector connecting C_α carbon atom of Glu59 with that of Lys79. $\vec{n}_a$ and $\vec{n}_b$ correspond to the radius and height directions, respectively. The height of GFP is determined by defining $\vec{n}_a$ as a vector that connected C_α carbon atom of Pro59 with that of Thr70. $\vec{n}_a$ and $\vec{n}_b$ correspond to the height and radius directions, respectively. These results are illustrated in Figure 1 in the main text.

SI-3 t-r dependent solution to thermal diffusion equation in the cylindrical model

We solve the thermal diffusion equation for the cylindrical model by ignoring angle (θ) and height (z) dependence. The differential equation is given below:[2,3]

$$\frac{\partial v}{\partial t} = D\left[\frac{\partial^2 v}{\partial r^2} + \frac{1}{r}\frac{\partial v}{\partial r}\right] \qquad (15)$$

Using the following transformation,

$$r = l\rho, t = \tau\chi$$

$$\tau = D^{-1}l^2 \qquad (16)$$

we can make eqn. 15 non-dimensional form, that is, eqn. 17 below. It is noted that *l* can be selected arbitrarily:

$$\frac{\partial v}{\partial \chi} = \frac{1}{\rho}\frac{\partial}{\partial \rho}\rho\frac{\partial v}{\partial \rho} \qquad (17)$$

If we consider a variable-separated solution,

$$v(\chi, \rho) = T(\chi)X(\rho) \qquad (18)$$

Eqn. 17 becomes

$$\frac{1}{T}\frac{\partial T}{\partial \chi} = \frac{1}{X}\frac{1}{\rho}\frac{\partial}{\partial \rho}\rho\frac{\partial X}{\partial \rho} = -\lambda^2 \qquad (19)$$

where λ denotes a real positive number. As for the function *T*, the solution is given as

$$T(\chi) = Ae^{-\lambda^2\chi} \qquad (20)$$

Meanwhile, function X satisfies the first-class Bessel differential equation:

$$\frac{\partial^2 X}{\partial \rho^2} + \frac{1}{\rho}\frac{\partial X}{\partial \rho} + \lambda^2 X = 0 \qquad (21)$$

Thus, we can give the solution to eqn. 17 as

$$v(t,r) = D\, exp\left(-\frac{\lambda^2 t}{\tau}\right) J_0\left(\lambda \cdot \frac{r}{l}\right) \quad (22)$$

It is noted that $J_0(\bullet)$ is the 0th order first-class Bessel function. Using eqn. 22, we can construct a general solution:

$$\frac{v(t,r)-v_f}{v_i-v_f} = \sum_{n=1}^{\infty} A_n\, exp\left(-\lambda_n{}^2 \frac{t}{\tau_n}\right) J_0\left(\lambda_n \frac{r}{l}\right) \qquad (23)$$

This is the normalized form of the solution to the thermal diffusion equation and the coefficients A_n can be given by considering an initial condition (*I.C.*) and boundary conditions (*B.C.*) as

$$I.C.\!:\; v(r,t=0) = v_i \quad (24)$$

$$B.C.\!:\; \kappa \frac{\partial v}{\partial r}\bigg|_{r=R_c} = -G\left(v - v_f\right) \quad (25)$$

Here, we suppose that the cylinder has radius of R_c and height of H_c.

The right-hand side of eqn. 23 is multiplied by $v_i - v_f$ and is substituted into *B.C.*:

$$left\, side\!:\; \kappa\left(v_f - v_i\right)\frac{\partial}{\partial r}\sum_{n=1}^{\infty} A_n\, exp\left(-\lambda_n{}^2 \frac{t}{\tau_n}\right) J_0\left(\lambda_n \frac{r}{l}\right)$$

$$= \kappa\left(v_f - v_i\right)\sum_{n=1}^{\infty} A_n\, exp\left(-\lambda_n{}^2 \frac{t}{\tau_n}\right)\frac{\partial}{\partial r} J_0\left(\lambda_n \frac{r}{l}\right)\bigg|_{r=R_c}$$

$$= -\kappa\left(v_f - v_i\right)\sum_{n=1}^{\infty} A_n \frac{\lambda_n}{l}\, exp\left(-\lambda_n{}^2 \frac{t}{\tau_n}\right) J_1\left(\lambda_n \frac{R_c}{l}\right) \quad (26)$$

$$right\, side\!:\, -G\left(v_f - v_i\right)\sum_{n=1}^{\infty} A_n\, exp\left(-\lambda_n{}^2 \frac{t}{\tau_n}\right) J_0\left(\lambda_n \frac{R_c}{l}\right) \quad (27)$$

Then, by combining the eqns. 26 and 27, we finally obtain

$$\frac{\lambda_n J_1\left(\lambda_n \frac{R_c}{l}\right)}{J_0\left(\lambda_n \frac{R_c}{l}\right)} = \frac{Gl}{\kappa} \equiv Bi \qquad (28)$$

By substituting eqn. 23 into *I.C.*, we obtain the following equation:

$$\frac{v(t=0,r)-v_f}{v_i-v_f} = 1 = \sum_{n=1}^{\infty} A_n \cdot J_0\left(\lambda_n \frac{r}{l}\right) \qquad (29)$$

Eqn. 29 is multiplied by $rJ_0\left(\lambda_m \frac{r}{l}\right)$ and integrated for r from 0 to R_c:

$$\frac{l^2}{{\lambda_m}^2} J_1\left(\frac{R_c}{l}\lambda_m\right) = l^2 \sum_{n=1}^{\infty} A_n \int_0^{\frac{R_c}{l}} dxxJ_0(\lambda_m x) J_0(\lambda_n x) \qquad (30)$$

On the right-hand side, if index m is not equal to index n, terms in the summation vanish due to the relation:

$$\int_0^{\frac{R}{l}} dx \cdot xJ_0(\lambda_m x) J_0(\lambda_n x) = \begin{cases} \frac{\lambda_n J_0\left(\lambda_m \frac{R_c}{l}\right) J_1\left(\lambda_n \frac{R_c}{l}\right) - \lambda_m J_0\left(\lambda_n \frac{R_c}{l}\right) J_1\left(\lambda_m \frac{R_c}{l}\right)}{\alpha_n - \alpha_m} & (m \neq n) \\ \frac{1}{2} J_0{}'\left(\lambda_m \frac{R_c}{l}\right)^2 + \frac{1}{2} J_0\left(\lambda_m \frac{R_c}{l}\right)^2 & (m = n) \end{cases} \qquad (31)$$

This relation can be obtained by combining the formula of the first-class Bessel function and eqn. 28, which can be confirmed easily. $J_1(\bullet)$ is the 1st order first-class Bessel function. According to the eqns. 30 and 31, the coefficient A_n has the following form:

$$A_n = \frac{2}{\lambda_n} \frac{J_1\left(\lambda_n \frac{R_c}{l}\right)}{J_0\left(\lambda_n \frac{R_c}{l}\right)^2 + J_1\left(\lambda_n \frac{R_c}{l}\right)^2} \qquad (32)$$

Here, we use R_c as l in eqn. 32 and substitute the A_n into eqn. 23, leading to

$$\frac{v(r,t)-v_f}{v_i-v_f} = \sum_{n=1}^{\infty} \frac{2}{\lambda_n} \cdot \frac{J_1(\lambda_n)}{J_0(\lambda_n)^2 + J_1(\lambda_n)^2} exp\left(-{\lambda_n}^2 \frac{t}{\tau}\right) J_0\left(\lambda_n \frac{r}{R}\right) \qquad (33)$$

By substituting this equation into the thermal diffusion equation dependent on time and radius, eqn. 15, we can obtain the relation between thermal diffusion coefficient and geometrical measure:

$D = \frac{R_c{}^2}{\tau}$ (34)

As in the case of the earlier study by Lervik,[1] we averaged eqn. 33 over the protein volume:

$$\frac{v(t) - v_f}{v_i - v_f} = \frac{1}{V_{cylinder}} \int_0^{R_c} rdr \int_0^{2\pi} d\theta \int_0^{\frac{H_c}{2}} dz \frac{v(r,t) - v_f}{v_i - v_f}$$

$$= \sum_{n=1}^{\infty} \frac{4}{\lambda_n{}^2} \frac{J_1(\lambda_n)^2}{J_0(\lambda_n)^2 + J_1(\lambda_n)^2} exp\left(-\lambda_n{}^2 \frac{t}{\tau_n}\right) \quad (35)$$

It is noted that $V_{cylinder}$ is $\pi R_c{}^2 H_c$. This is the formula for simulation data fitting, referred to as eqn. 3a in the main text, by retaining only the first term (n = 1) in the n series which is expected to decay rapidly as n increases.

SI-4 t-z dependent solution to thermal diffusion equation in the cylindrical model

We solve the thermal diffusion equation for the cylindrical model by ignoring angle (θ) and radius (r) dependence. The differential equation is given below:

$$\frac{\partial v}{\partial t} = D\left[\frac{\partial^2 v}{\partial z^2}\right] \qquad (36)$$

Using the following transformation,

$$z = l\zeta,\ t = \tau\chi,\ \tau = D^{-1}l^2 \qquad (37)$$

we can make eqn. 36 a non-dimensional form, that is, eqn. 38 below. It is noted that *l* can be selected arbitrarily:

$$\frac{\partial v}{\partial \chi} = \frac{\partial^2 v}{\partial \zeta^2} \qquad (38)$$

If we consider a variable-separated solution,

$$v(\chi,\zeta) = T(\chi)X(\zeta) \qquad (39)$$

eqn. 38 becomes

$$\frac{1}{T}\frac{\partial T}{\partial \chi} = \frac{1}{X}\frac{\partial^2 X}{\partial \zeta^2} = -\lambda^2 \qquad (40)$$

where λ denotes a real positive number. As for the function *T*, the solution is given as

$$T(\chi) = Ae^{-\lambda^2\chi} \quad (41)$$

Meanwhile, function *X* satisfies

$$\frac{\partial^2 X}{\partial \zeta^2} = -\lambda^2 X \qquad (42)$$

and the solution is

$X(\zeta) = B\,cos(\lambda\zeta - \phi)$ (43)

Thus, we can give the solution to eqn. 36 as

$v(t, z) = A \cdot e^{-\frac{\lambda^2 t}{\tau}} \cdot cos\left(\lambda \frac{z}{l} - \phi\right)$ (44)

It is noted that ϕ can be set to 0 due to the inversion symmetry of cylinder around the origin so that we use the following form hereafter:

$v(t, z) = A \cdot e^{-\frac{\lambda^2 t}{\tau}} \cdot cos\left(\lambda \frac{z}{l}\right)$ (45)

Using eqn. 45, we can construct a general solution:

$\frac{v(t,z) - v_f}{v_i - v_f} = \sum_{n=1}^{\infty} A_n \cdot exp\left(-\frac{{\lambda_n}^2 t}{\tau}\right) \cdot cos\left(\lambda_n \frac{z}{l}\right)$ (46)

This is the normalized form of the solution to the thermal diffusion equation and the coefficients A_n can be given by considering an initial condition (*I.C.*) and boundary conditions (*B.C.*) as

$I.C.:\ v(r, t = 0) = v_i$ (47)

$B.C.:\ \kappa \frac{\partial v}{\partial z}\Big|_{z=\frac{H_C}{2}} = -G(v - v_f),\ \kappa \frac{\partial v}{\partial z}\Big|_{z=-\frac{H_C}{2}} = -G(v - v_f)$ (48)

We suppose that the cylinder has radius of R_c and height of H_c. Due to the inversion symmetry of cylinder around the origin, it is enough to consider either of the two boundary conditions (we here consider the left one given in eqn. 48).

The right-hand side of eqn. 46 is multiplied by $v_i - v_f$ and is substituted into *B.C.*:

$$left\ side:\ \kappa\left(v_f - v_i\right)\frac{\partial}{\partial z}\left[\sum_{n=1}^{\infty} A_n \cdot exp\left(-\frac{{\lambda_n}^2 t}{\tau}\right)\cdot cos\left(\lambda_n \frac{z}{l}\right)\right]_{z=\frac{H_c}{2}}$$

$$= \kappa\left(v_f - v_i\right)\sum_{n=1}^{\infty} A_n \cdot exp\left(-\frac{{\lambda_n}^2 t}{\tau}\right)\left[\frac{\partial}{\partial z} cos\left(\lambda_n \frac{z}{l}\right)\right]_{z=\frac{H_c}{2}}$$

$$= -\frac{\lambda_n}{l}\kappa\left(v_f - v_i\right)\textstyle\sum_{n=1}^{\infty} A_n \cdot exp\left(-\frac{{\lambda_n}^2 t}{\tau}\right) sin\left(\lambda_n \frac{1}{l}\frac{H_c}{2}\right) \qquad (49)$$

$$right\ side:\ -G\left(v_f - v_i\right)\textstyle\sum_{n=1}^{\infty} A_n \cdot exp\left(-\frac{{\lambda_n}^2 t}{\tau}\right)\cdot cos\left(\lambda_n \frac{H_c/2}{l}\right) \qquad (50)$$

Then, by combining eqns. 49 and 50, we finally obtain

$$\lambda_n\, tan\left(\lambda_n \frac{1}{l}\frac{H_c}{2}\right) = \frac{G_\perp}{\kappa_\perp} l = Bi \qquad (51)$$

By substituting eqn. 46 into *I.C.*, we obtain the following equation:

$$\frac{v(t=0,z)-v_f}{v_i - v_f} = 1 = \textstyle\sum_{n=1}^{\infty} A_n \cdot cos\left(\lambda_n \frac{z}{l}\right) \qquad (52)$$

Eqn. 52 is multiplied by $cos\frac{\lambda_m}{l}z$ and integrated for *z* from 0 to $H_C/2$, then leading to

$$\int_0^{\frac{H_c}{2}} cos\left(\lambda_m \cdot \frac{z}{l}\right) dz = \int_0^{\frac{H_c}{2}} A_m \cdot cos\left(\lambda_m \cdot \frac{z}{l}\right)\cdot cos\left(\lambda_m \cdot \frac{z}{l}\right)\cdot dz \qquad (53)$$

On the right-hand side, if index *m* was not equal to index *n*, terms in the summation vanished due to the combination of eqn. 51 with the following relation,

$$\int_0^{\frac{H_c}{2}} cos\frac{\lambda_m}{l}z \cdot cos\frac{\lambda_n}{l}z dz = \frac{l}{{\lambda_m}^2 - {\lambda_n}^2}\left[\lambda_m\, sin\frac{\lambda_m}{l}\frac{H_c}{2}\cdot cos\frac{\lambda_n}{l}\frac{H_c}{2} - \lambda_n\, sin\frac{\lambda_m}{l}\frac{H_c}{2}\cdot cos\frac{\lambda_n}{l}\frac{H_c}{2}\right]$$

(54)

According to eqn. 53, the coefficient A_n has the following form:

$$A_n = \frac{4\cdot sin\left(\lambda_n \cdot \frac{1}{l}\cdot\frac{H_c}{2}\right)}{\left(2\cdot\lambda_n \cdot \frac{H_c}{2}\cdot\frac{1}{l} + sin\left(2\cdot\lambda_n \cdot \frac{1}{l}\cdot\frac{H_c}{2}\right)\right)} \qquad (55)$$

Here, we use $H_c/2$ as *l* in eqn. 55 and substitute the A_n into eqn. 46, then leading to

$$\frac{v(t,z)-v_f}{v_i-v_f} = \sum_{n=1}^{\infty} \frac{4 \cdot sin(\lambda_n)}{(2 \cdot \lambda_n + sin(2 \cdot \lambda_n))} \cdot exp\left(-\frac{{\lambda_n}^2 \cdot t}{\tau}\right) \cdot cos\left(\lambda_n \cdot \frac{z}{\frac{H_c}{2}}\right) \qquad (56)$$

By substituting this equation into the thermal diffusion equation dependent on time and height, eqn. 36, we can obtain the relation between thermal diffusion coefficient and geometrical measure:

$$D = \frac{{H_c}^2}{4\tau} \qquad (57)$$

As in the case of the earlier study by Lervik,[1] we averaged eqn. 56 over the protein volume:

$$\frac{v(t)-v_f}{v_i-v_f} = \frac{1}{V_{cylinder}} \int_0^{R_c} r d\,r \int_0^{2\pi} d\theta \int_0^{\frac{H_c}{2}} dz \frac{v(t,z)-v_f}{v_i-v_f}$$

$$= \sum_{n=1}^{\infty} \frac{4 \cdot sin^2(\lambda_n)}{\lambda_n \cdot (2 \cdot \lambda_n + sin(2 \cdot \lambda_n))} \cdot exp\left(-\frac{{\lambda_n}^2 \cdot t}{\tau}\right) \qquad (58)$$

It is noted that $V_{cylinder}$ is $\pi {R_c}^2 H_c$. This is the formula for simulation data fitting, referred to as eqn. 3b in the main text by retaining only the first term (n = 1) in the n series which is expected to decay rapidly as n increases.

SI-5 t-r-z dependent solution to thermal diffusion equation in the cylindrical model

We solve the thermal diffusion equation for the cylindrical model with the radius R_c and height H_c by ignoring angle (θ) dependence:

$$\frac{\partial v}{\partial t} = D_{//}\left[\frac{\partial^2 v}{\partial r^2} + \frac{1}{r}\frac{\partial v}{\partial r}\right] + D_{\perp}\frac{\partial^2 v}{\partial z^2} \quad (59)$$

Using the solutions to the t-r and t-z dependent equations as illustrated in the preceding sections, we can construct a general solution as

$$\frac{v(r,z,t)-v_f}{v_i-v_f} = \sum_{m=1}^{\infty}\sum_{n=1}^{\infty} A_{mn} J_0\left(\lambda_m \frac{r}{R_c}\right) cos\left(\mu_n \frac{z}{\frac{H_c}{2}}\right) exp\left[-\left(\frac{\lambda_m{}^2}{\tau_{\parallel}} + \frac{\mu_n{}^2}{\tau_{\perp}}\right) t\right] \quad (60)$$

Eqn. 60 is the normalized form of the solution to the thermal diffusion equation, eqn. 59, and the coefficients A_{mn} can be given by considering the initial condition (*I.C.*) and boundary conditions (*B.C.*) as

$$I.C.:\ v(r, t = 0) = v_i \quad (61)$$

$$B.C.:\ \kappa_{\parallel} \left.\frac{\partial v}{\partial r}\right|_{r=R_c} = -G_{\parallel}\left(v(r = R_c, z, t) - v_f\right) \quad (62)$$

$$\kappa_{\perp} \left.\frac{\partial v}{\partial z}\right|_{z=\frac{H_c}{2}} = -G_{\perp}\left(v\left(r, z = \frac{H_c}{2}, t\right) - v_f\right),\ \kappa_{\perp} \left.\frac{\partial v}{\partial z}\right|_{z=-\frac{H_c}{2}} = -G_{\perp}\left(v(r, z = -\frac{H_c}{2}, t) - v_f\right) \quad (63)$$

The arguments in the exponential term (for τs in eqn. 60), thermal conductivity and thermal conductance are considered for both the radius and height directions, which are annotated by // and ⊥, respectively. We suppose that the cylinder has radius of R_c and height of H_c. Due to the inversion symmetry of cylinder around the origin, it is enough to

consider either of the two boundary conditions for the height direction (we here consider the left one given in eqn. 63).

The right-hand side of eqn. 60 is multiplied by $v_i - v_f$ and is substituted into *B.C.* for the radius direction:

left side: $\kappa_{\parallel}(v_f - v_i)\frac{\partial}{\partial r}\sum_{m=1}^{\infty}\sum_{n=1}^{\infty} A_{mn} J_0\left(\lambda_m \frac{r}{R_c}\right) cos\left(\mu_n \frac{z}{\frac{H_c}{2}}\right) exp\left[-\left(\frac{\lambda_m{}^2}{\tau_{\parallel}} + \frac{\mu_n{}^2}{\tau_{\perp}}\right)t\right]$

$$= \kappa_{\parallel}(v_f - v_i)\sum_{m=1}^{\infty}\sum_{n=1}^{\infty} A_{mn}\, exp\left[-\left(\frac{\lambda_m{}^2}{\tau_{\parallel}} + \frac{\mu_n{}^2}{\tau_{\perp}}\right)t\right] cos\left(\mu_n \frac{z}{\frac{H_c}{2}}\right)\frac{\partial}{\partial r} J_0\left(\lambda_n \frac{r}{R_c}\right)\Bigg|_{r=R_c}$$

$$= -\kappa_{\parallel}(v_f - v_i)\sum_{m=1}^{\infty}\sum_{n=1}^{\infty}\frac{\lambda_n}{R_c} A_{mn}\, exp\left[-\left(\frac{\lambda_m{}^2}{\tau_{\parallel}} + \frac{\mu_n{}^2}{\tau_{\perp}}\right)t\right] cos\left(\mu_n \frac{z}{\frac{H_c}{2}}\right) J_1\left(\lambda_n \frac{r}{R_c}\right)\Bigg|_{r=R_c}$$

$$= -\kappa_{\parallel}(v_f - v_i)\sum_{m=1}^{\infty}\sum_{n=1}^{\infty}\frac{\lambda_n}{R_c} A_{mn}\, exp\left[-\left(\frac{\lambda_m{}^2}{\tau_{\parallel}} + \frac{\mu_n{}^2}{\tau_{\perp}}\right)t\right] cos\left(\mu_n \frac{z}{\frac{H_c}{2}}\right) J_1(\lambda_n) \quad (64)$$

right side: $-G_{\parallel}(v_f - v_i)\sum_{m=1}^{\infty}\sum_{n=1}^{\infty} A_{mn} J_0(\lambda_m)\cos\left(\mu_n \frac{z}{\frac{H_c}{2}}\right) exp\left[-\left(\frac{\lambda_m{}^2}{\tau_{\parallel}} + \frac{\mu_n{}^2}{\tau_{\perp}}\right)t\right]$ (65)

Then, by combining the above eqns. 64 and 65, we obtain

$$\frac{\lambda_n J_1(\lambda_n)}{J_0(\lambda_n)} = \frac{G_{\parallel} R_c}{\kappa_{\parallel}} \equiv Bi_{\parallel} \quad (66)$$

Similarly, the right-hand side of eqn. 60 is multiplied by $v_i - v_f$ and is substituted into *B.C.* for the height direction:

left side: $\kappa_{\perp}(v_f - v_i)\frac{\partial}{\partial z}\sum_{m=1}^{\infty}\sum_{n=1}^{\infty} A_{mn} J_0\left(\lambda_m \frac{r}{R_c}\right) cos\left(\mu_n \frac{z}{\frac{H_c}{2}}\right) exp\left[-\left(\frac{\lambda_m{}^2}{\tau_{\parallel}} + \frac{\mu_n{}^2}{\tau_{\perp}}\right)t\right]\Bigg|_{z=\frac{H_c}{2}}$

$$= \kappa_{\perp}(v_f - v_i)\sum_{m=1}^{\infty}\sum_{n=1}^{\infty} A_{mn} J_0\left(\lambda_m \frac{r}{R_c}\right)\frac{\partial}{\partial z} cos\left(\mu_n \frac{z}{\frac{H_c}{2}}\right)\Bigg|_{z=\frac{H_c}{2}} exp\left[-\left(\frac{\lambda_m{}^2}{\tau_{\parallel}} + \frac{\mu_n{}^2}{\tau_{\perp}}\right)t\right]$$

$$= -\frac{\mu_n}{\frac{H_c}{2}}\kappa_{\perp}(v_f - v_i)\sum_{m=1}^{\infty}\sum_{n=1}^{\infty} A_{mn} J_0\left(\lambda_m \frac{r}{R_c}\right)\sin(\mu_n)\, exp\left[-\left(\frac{\lambda_m{}^2}{\tau_{\parallel}} + \frac{\mu_n{}^2}{\tau_{\perp}}\right)t\right] \quad (67)$$

$$right\ \ side:\ \ -G_{\perp}(v_f - v_i)\sum_{m=1}^{\infty}\sum_{n=1}^{\infty}A_{mn}J_0\left(\lambda_m\frac{r}{R}\right)cos\left(\mu_n\frac{z}{\frac{H_c}{2}}\right)exp\left[-\left(\frac{{\lambda_m}^2}{\tau_{\parallel}}+\frac{{\mu_n}^2}{\tau_{\perp}}\right)t\right]$$

(68)

Then, by combining eqns. 67 and 68 above, we obtain

$$\mu_n\, tan(\mu_n) = \frac{G_{\perp}}{\kappa_{\perp}}\cdot\frac{H_c}{2} \equiv Bi_{\perp} \qquad (69)$$

By substituting eqn. 60 into *I.C.*, we obtain the following equation:

$$\frac{v(r,z,t=0)-v_f}{v_i-v_f} = 1 = \sum_{m=1}^{\infty}\sum_{n=1}^{\infty}A_{mn}J_0\left(\lambda_m\frac{r}{R_c}\right)cos\left(\mu_n\frac{z}{\frac{H_c}{2}}\right) \qquad (70)$$

Eqn. 70 is operated by $\int_0^{R_c} rJ_0\left(\lambda_{m'}\frac{r}{R_c}\right)$ with the integration of r from 0 to R_c, then leading to

$$\sum_{n=1}^{\infty}A_{mn}\,cos\left(\mu_n\frac{z}{\frac{H_c}{2}}\right) = \frac{2}{\lambda_m}\frac{J_1(\lambda_m)}{J_0{}'(\lambda_m)^2 + J_0(\lambda_m)^2}$$

$$= \frac{2}{\lambda_m}\frac{J_1(\lambda_m)}{J_0(\lambda_m)^2+J_1(\lambda_m)^2} \qquad (71)$$

by recalling the following formulas:

$$\int_0^1 dxxJ_0(\lambda_m x)J_0(\lambda_{m'}x) = \begin{cases}\frac{\lambda_{m'}J_0(\lambda_m)J_1(\lambda_{m'})-\lambda_m J_0(\lambda_{m'})J_1(\lambda_m)}{{\lambda_{m'}}^2-{\lambda_m}^2} & (m \neq m')\\ \frac{1}{2}J_0{}'(\lambda_m)^2+\frac{1}{2}J_0(\lambda_m)^2 & (m = m')\end{cases} \qquad (72)$$

Furthermore, eqn. 71 is multiplied by $\cos\left(\mu_n\frac{z}{\frac{H_c}{2}}\right)$ and integrated for z from 0 to $H_c/2$. By considering eqn. 69 and

$$\int \cos\frac{\mu_n}{\frac{H_c}{2}}z\cdot\cos\frac{\mu_m}{\frac{H_c}{2}}zdz = \frac{\left(\frac{H_c}{2}\right)^2}{{\mu_n}^2-{\mu_m}^2}\left[\frac{\mu_n}{\frac{H_c}{2}}\sin\frac{\mu_n}{\frac{H_c}{2}}z\cdot\cos\frac{\mu_m}{\frac{H_c}{2}}z - \frac{\mu_m}{\frac{H_c}{2}}\sin\frac{\mu_m}{\frac{H_c}{2}}z\cdot\cos\frac{\mu_n}{\frac{H_c}{2}}z\right]$$

$$= \frac{\frac{H_c}{2}}{{\mu_n}^2-{\mu_m}^2}\left[\mu_n\sin\frac{\mu_n}{\frac{H_c}{2}}z\cdot\cos\frac{\mu_m}{\frac{H_c}{2}}z - \mu_m\sin\frac{\mu_m}{\frac{H_c}{2}}z\cdot\cos\frac{\mu_n}{\frac{H_c}{2}}z\right] \qquad (73)$$

we finally obtain

$$A_{mn} = 4 \cdot \frac{sin(\mu_n)}{(2\mu_n + sin(2\mu_n))} \cdot \frac{2}{\lambda_m} \frac{J_1(\lambda_m)}{J_0(\lambda_m)^2 + J_1(\lambda_m)^2} \qquad (74)$$

By substituting eqn. 60 into eqn. 59, we obtain the following relations between the thermal diffusion constants and the relaxation times:

$$D_{\parallel} = \frac{{R_c}^2}{\tau_{\parallel}} \qquad (75)$$

$$D_{\perp} = \frac{\left(\frac{H_c}{2}\right)^2}{\tau_{\perp}} \qquad (76)$$

The thermal conductivities for the radial and longitudinal (vertical) directions are then given by

$$\kappa_{//} = (C_p/V)D_{//} \qquad (77)$$

$$\kappa_{\perp} = (C_p/V)D_{\perp} \qquad (78)$$

respectively, where C_p and $V = \pi R_c^2 H_c$ refer to the heat capacity and the volume of cylindrical protein, respectively. While the simultaneous determination of the thermal conductivities and conductances for both the radial and longitudinal directions through a single MD simulation is possible in principle, its practical execution is difficult due to the reduction in numerical reliability compared to the cases in which the thermal conductions along the two directions are considered individually.

REFRERENCES

1. Lervik A., Bresme F., Kjelstrup S., Bedeaux D., Rubi J. M. Heat transfer in protein-water interfaces. *Phys. Chem. Chem. Phys.* **2010**, 12, 1610-1617.

2. Incropera F.P., DeWitt D.P. *Fundamentals of Heat and Mass Transfer*, 3rd ed., Wiley, New York, 1990.

3. Carslaw H.S, Jaeger J.C. *Conduction of Heat in Solids*, 2nd ed., Oxford University Press, New York, 2011.
